# Economic burden and influence factors among hospitalized children with bronchiolitis or pneumonia: a multiregional study in China

**DOI:** 10.3389/fpubh.2024.1364854

**Published:** 2024-09-02

**Authors:** Hongfei Hu, Ting Zhou, Junyang Gao, Yanglu Ou, Aixia Ma, Pei Wang

**Affiliations:** ^1^School of International Pharmaceutical Business, China Pharmaceutical University, Nanjing, China; ^2^School of Public Health, Fudan University, Shanghai, China; ^3^Key Lab of Health Technology Assessment, National Health Commission of the People’s Republic of China (Fudan University), Shanghai, China

**Keywords:** bronchiolitis, pneumonia, Chinese pediatrics, economic burden, influential factors

## Abstract

**Background:**

Bronchiolitis and pneumonia are both significant lower respiratory tract infections with a profound impact on children’s health. The purpose of this study is to explore the economic burden and related influence factors of pediatric patients with bronchiolitis and pneumonia in China.

**Methods:**

A face-to-face interview was employed for the investigation of hospitalized patients (≤5 years old) with bronchiolitis and pneumonia, along with their guardians from January to October 2019. Demographic and costs were collected from Shanghai, Zhengzhou, and Kunming, representing three regions with different levels of economic development in China. Multiple linear regression analysis was used to explore factors associated with the economic burden of the diseases.

**Results:**

A total of 338 patients with bronchiolitis and 529 patients with pneumonia were included in the analysis. The average hospitalization and total cost for patients with bronchiolitis are 4,162 CNY and 5,748 CNY, respectively, while those with pneumonia are 6,096 CNY and 7,783 CNY. Patients from Shanghai, both bronchiolitis and pneumonia, exhibited the lowest cost expenditures, with average total costs of 3,531 CNY and 3,488 CNY, respectively. Multiple regression analysis indicated that, among bronchiolitis patients, factors such as region, medical insurance, relationship, loss of work time, and length of stay were found to be significantly associated with both hospitalization cost and total cost (*p* < 0.05). For pneumonia patients, the hospitalization cost and total cost were significantly impacted by region, medical insurance, and length of stay (*p* < 0.05).

**Conclusion:**

Bronchiolitis and pneumonia in children put substantial economic burden on families of affected children. The financial strain varies significantly across different regions, with families in underdeveloped areas and those dealing with pneumonia facing particularly daunting challenges. Targeted policies to reduce healthcare costs and improve insurance coverage, especially in economically disadvantaged regions are needed.

## Introduction

Bronchiolitis and pneumonia are common lower respiratory tract infections impacting children’s well-being (1). They can manifest symptoms like fever, cough, and respiratory distress (1–3). In severe instances, these conditions may result in fatal outcomes, presenting substantial challenges to global public health.

Bronchiolitis, marked by inflammation of the small airways in the lungs due to viral infections, predominantly affects children under 2 years old, emerging as a primary cause for hospitalization (2, 4). In Canada, the mortality rate attributed to bronchiolitis stands at 2.8 per 100,000 person-years (5). Notably, hospitalization rates were 14.0 and 12.7 per 1,000 person-years during 2004–2005 and 2017–2018, respectively, with a noteworthy increase in intensive care unit (ICU) admissions from 38.1 to 87.8 per 1,000 person-years (5). The gravity of bronchiolitis is also evident in China, where evidence reveals that children with bronchiolitis constitute 14.57% of total hospitalizations for respiratory diseases, with the highest proportion among hospitalized children under the age of 1 (6).

Pneumonia denotes the infection of small air sacs (alveoli) and surrounding tissues deep within the lungs, primarily triggered by viral and bacterial infections (3). It stands as a major contributor to child mortality, responsible for approximately one million deaths annually and representing 14.9% of fatalities among children under the age of 5 in global (7). Notably, China exhibits one of the highest incidence rates of pneumonia in children. Among children under 5 years old in China, the incidence rate of pneumonia is 2.55%, with a hospitalization rate of 1.40% (8). Despite a substantial decline in pneumonia-related deaths from 2000 to 2013, it still constitutes 14.6% of the total deaths in children under 5 years old (9).

The gravity of the illnesses also impose a significant economic burden on families. To mitigate the global mortality rate of bronchiolitis, there is a consistent upward trend in the utilization and cost of intensive care resources (10). In China, a retrospective analysis covering the period from 2015 to 2018 revealed that, for 169 children with bronchiolitis in Gansu Province, the average length of hospital stay was 8.5 days, and the average hospitalization cost per person was 7,470 Chinese Yuan (CNY) (11). In low-and middle-income countries, the direct medical expenses for severe pneumonia can represent a substantial portion, ranging from 26.6 to 115.8% of the monthly household income of patients (12). The burden on children under the age of 5 in China, particularly infants with pneumonia, is enormous. Several studies have assessed its economic burden in China (8, 11, 13, 14). An analysis of 149 children with pneumonia in Shanghai indicated the average hospitalization cost was 7,623 CNY, surpassing the regional average in Asian countries (14). The average length of hospital stay, at 7.9 days, exceeds both the World Health Organization’s recommended 5 days and the latest global review estimate of 5.8 days for pediatric pneumonia treatment systems (14).

Although the studies have explored the economic burden of patients with bronchiolitis and pneumonia in China, most studies were limited to one region or focused on one respiratory disease. Therefore, this study aimed to investigate the economic burden of patients with bronchiolitis and pneumonia in three cities representing different levels of development in China and explore the influence factors of economic burden differences, in order to update and supplement relevant disease economic burden data.

## Methods

### Study design

We conducted face-to-face interviews in pediatric patients with pneumonia or bronchiolitis from January to October 2019. Three provincial capital cities, Shanghai, Zhengzhou and Kunming were selected according to the economic status and geographical location in China. These cities are located in the eastern, central, and western regions respectively, representing high, medium, and low economic levels. The study sites in each city were chosen to be tertiary general hospitals or specialized children’s hospitals. This study adhered to the ethical guidelines set forth in the Declaration of Helsinki and obtained approval from the Institutional Review Board of the School of Public Health at Fudan University (IRB00002408).

### Patient population

The subjects of the survey were recruited after being introduced by doctors in sampled hospitals. The inclusion criteria for pediatric patients included that these patients were hospitalized children under the age of 5, clinically diagnosed with pneumonia or bronchiolitis, and had no concomitant diseases. The exclusion criteria for pediatric patients were children admitted to the Intensive Care Unit. The guardians of eligible patients were invited to participate in the study. The inclusion criteria for the guardians included that they were primary caregivers and able to understand the questionnaire. If patients and their guardians fail to satisfy the inclusion criteria, they will be excluded. All patients/guardians provided informed consent before enrollment. Participants who completed the survey would receive a 50 CNY voucher as an incentive.

### Collection of data

In this study, trained researchers conducted two face-to-face interviews with pediatric patients and their guardians. The first interview took place within the initial 2 days following hospitalization. A questionnaire was used to collect data on the sociodemographic characteristics of them. For the patients, this encompassed details such as age, sex, and medical insurance status. As for the guardians, their sociodemographic characteristics included age, sex, relationship with the child, education level, marital status, employment, monthly income level, ethnicity, residence type and religious status. The second interview was conducted at the time of the child’s discharge, and hospitalization costs, out-of-pocket costs, traffic costs, time lost from work, and length of hospital stay were recorded.

### Assessment of economic burden

In addition to the economic burden data directly obtained from the questionnaire, including hospitalization costs, transportation costs, etc., we also calculated both the total and indirect costs associated with each specific disease. The total costs included hospitalization costs, traffic costs and indirect costs (loss of caregiver productivity). The indirect costs were estimated employing the human capital approach, calculated by multiplying the loss of work time including before admission and during the hospitalization by the hourly wage. The hourly wage was derived from the latest published 2022 annual average income in China (based on 8 h per work day and 250 work days per year) (15, 16). It is important to note that the hospitalization cost was defined as the direct medical costs, including out-of-pocket costs and insurance costs (if any). All cost data have been adjusted to 2023 CNY using China’s annual consumer price index (CPI) (17).

### Statistical analysis

Descriptive analysis was performed to present the sociodemographic characteristics and economic burden data. Wilcoxon-Mann–Whitney test and Kruskal–Wallis *H* test was used for continuous variables to compare the differences between groups after normality test. Continuous variables were presented as the mean and standard deviation (SD), as well as median and interquartile range (IQR). Categorical data were presented in numbers and percentages, and the differences between groups were compared by Chi-square tests. Moreover, in instances where the Kruskal-Wallis tests demonstrated statistical significance, *post hoc* analyses were conducted using kwAllPairsDunn’s test with Bonferroni correction to compare differences in pairwise groups (18).

Multiple linear regression was used to analyze the factors influencing the economic burden of children with pneumonia or bronchiolitis. In the model, the dependent variables were the natural logarithmic transformation of hospitalization costs and total costs. The independent variables included all sociodemographic characteristics of children and their guardians, lost work time for guardians and length of hospital stay. The multiple stepwise regression analysis was performed using the forward and backward entry method. The goodness-of-fit of the regression model was assessed by presenting *R*-square values, and collinearity among influencing factors was diagnosed using the variance inflation factor (19). Missing data were excluded from analysis.

For all statistical analyses, R software (version 4.3.1) was utilized. A two-tailed *p*-value less than 0.05 was considered statistically significant.

## Results

### Characteristics of study subjects

A total of 338 patients with bronchiolitis and 529 patients with pneumonia were included in the analysis. In the bronchiolitis cohort, there were 128, 120, and 90 patients from Shanghai, Zhengzhou, and Kunming, respectively. Boys constituted 52.1% (176/338) of the patients, while the majority of guardians were female (62.1%). The median (IQR) age of the patients was 2.79 (1.08–4.00) years old and the guardians were 32.0 (29.0–35.0) years old. Approximately 36.1% (122/338) of the guardians reported not using any medical insurance. Most guardians were the mothers of the children (60.4%) and had received a university education or above (46.4%). Moreover, 95.0% (321/338) of the guardians were married, 47.3% (160/338) worked in enterprises or were self-employed, 96.4% (326/338) were Han nationality (97.0%), 63.6% (215/338) earned over 10,000 CNY per month, 61.5% lived in cities (63.5%) and 97.3% (328/338) were not religious. The characteristics of the patients and their guardians were statistically different among the three regions except for patient sex (*p* = 0.107) (Table 1).

**Table 1:** Sociodemographic characteristics of the children with bronchiolitis and their guardians in three regions.

Characteristics	Total	Shanghai	Zhengzhou	Kunming	*p*-value^a^
	*N* = 338	*N* = 128	*N* = 120	*N* = 90	
Sex					0.109
Boys	176 (52.1%)	65 (50.8%)	56 (46.7%)	55 (61.1%)	
Girls	162 (47.9%)	63 (49.2%)	64 (53.3%)	35 (38.9%)	
Age (year)					<0.001
Mean (SD)	2.85 (1.85)	2.02 (1.32)	2.70(1.25)	4.22 (2.35)	
Median (IQR)	2.79 (1.08–4.00)	1.10 (1.00–3.00)	2.80 (1.70–3.70)	4.20 (2.50–4.80)	
Medical insurance					<0.001
Yes	153 (45.3%)	90 (70.3%)	37 (30.8%)	26 (28.9%)	
No	122 (36.1%)	38 (29.7%)	82 (68.3%)	2 (2.2%)	
Unknown/refuse to answer	63 (18.6%)	0 (0.0%)	1 (0.8%)	62 (68.9%)	
Guardian’s age (year)					0.008
Mean (SD)	33.17 (7.79)	35.40 (10.87)	32.40 (3.30)	31.01 (5.80)	
Median (IQR)	32.0 (29.0–35.0)	31.5 (29.0–36.2)	32.0 (30.0–35.0)	30.0 (28.2–33.0)	
Guardian’s sex					0.019
Male	128 (37.9%)	41 (32.0%)	42 (35.0%)	45 (50.0%)	
Female	210 (62.1%)	87 (68.0%)	78 (65.0%)	45 (50.0%)	
Relationship					<0.001
Father	117 (34.6%)	27 (21.1%)	42 (35.0%)	48 (53.3%)	
Mother	204 (60.4%)	84 (65.6%)	78 (65.0%)	42 (46.7%)	
Others^b^	17 (5.0%)	17 (13.3%)	0 (0.0%)	0 (0.0%)	
Education level					<0.001
Uneducated	4 (1.2%)	1 (0.8%)	0 (0.0%)	3 (3.3%)	
Primary school	17 (5.0%)	5 (3.9%)	1 (0.8%)	11 (12.2%)	
Junior high school	62 (18.3%)	6 (4.7%)	1 (0.8%)	55 (61.1%)	
Senior high school	54 (16.0%)	21 (16.4%)	16 (13.3%)	17 (18.9%)	
Junior college	42 (12.4%)	14 (10.9%)	27 (22.5%)	1 (1.1%)	
University and above	157 (46.4%)	81 (63.3%)	75 (62.5%)	1 (1.1%)	
Unknown/refuse to answer	2 (0.6%)	0 (0.0%)	0 (0.0%)	2 (2.2%)	
Marriage status					0.004
Single	5 (1.5%)	2 (1.6%)	0 (0.0%)	3 (3.3%)	
Married	321 (95.0%)	123 (96.1%)	118 (98.3%)	80 (88.9%)	
Separated	5 (1.5%)	1 (0.8%)	1 (0.8%)	3 (3.3%)	
Widowhood	3 (0.9%)	2 (1.6%)	1 (0.8%)	0 (0.0%)	
Refused to answer	4 (1.2%)	0 (0.0%)	0 (0.0%)	4 (4.4%)	
Employment					<0.001
Enterprise/self-employed	160 (47.3%)	56 (43.8%)	78 (65.0%)	26 (28.9%)	
Full-time student	2 (0.6%)	1 (0.8%)	0 (0.0%)	1 (1.1%)	
Government/institution employee	78 (23.1%)	33 (25.8%)	35 (29.2%)	10 (11.1%)	
Full-time stay at home/housewife	43 (12.7%)	23 (18.0%)	3 (2.5%)	17 (18.9%)	
Retire	8 (2.4%)	8 (6.2%)	0 (0.0%)	0 (0.0%)	
Farming	31 (9.2%)	6 (4.7%)	1 (0.8%)	24 (26.7%)	
Temporary worker	10 (3.0%)	0 (0.0%)	3 (2.5%)	7 (7.8%)	
Unemployed	4 (1.2%)	1 (0.8%)	0 (0.0%)	3 (3.3%)	
Unknown/refuse to answer	2 (0.6%)	0 (0.0%)	0 (0.0%)	2 (2.2%)	
Monthly income (CNY)					<0.001
<5,000	64 (18.9%)	1 (0.8%)	3 (2.5%)	60 (66.7%)	
5,000-10,000	45 (13.3%)	0 (0.0%)	21 (17.5%)	24 (26.7%)	
10,000-20,000	55 (16.3%)	1 (0.8%)	49 (40.8%)	5 (5.6%)	
20,000-30,000	50 (14.8%)	18 (14.1%)	32 (26.7%)	0 (0.0%)	
30,000-50,000	60 (17.8%)	48 (37.5%)	12 (10.0%)	0 (0.0%)	
>50,000	50 (14.8%)	47 (36.7%)	3 (2.5%)	0 (0.0%)	
Unknown/refuse to answer	14 (4.1%)	13 (10.2%)	0 (0.0%)	1 (1.1%)	
Guardian’s ethnicity					<0.001
Han	326 (96.4%)	128 (100.0%)	120 (100.0%)	78 (86.7%)	
Others	12 (3.6%)	0 (0.0%)	0 (0.0%)	12 (13.3%)	
Residence type					<0.001
City	208 (61.5%)	90 (70.3%)	93 (77.5%)	25 (27.8%)	
Town	53 (15.7%)	24 (18.8%)	19 (15.8%)	10 (11.1%)	
Rural	76 (22.5%)	14 (10.9%)	8 (6.7%)	54 (60.0%)	
Unknown/refuse to answer	1 (0.3%)	0 (0.0%)	0 (0.0%)	1 (1.1%)	
Religious status					0.039
No	328 (97.0%)	127 (99.2%)	117 (97.5%)	84 (93.3%)	
Yes	7 (2.1%)	1 (0.8%)	3 (2.5%)	3 (3.3%)	
Unknown/refuse to answer	3 (0.9%)	0 (0.0%)	0 (0.0%)	3 (3.3%)	

SD, standard deviations; IQR, interquartile range; CNY: Chinese Yuan. ^a^Comparison of population characteristics among the three regions. Categorical variables and continuous variables were analyzed using chi-square test and Kruskal–Wallis *H* test, respectively.

^b^This refers to a guardian such as a non-parental relative of the affected child.

For pneumonia patients, there were 173, 120, and 236 patients from the three cities, respectively. The median (IQR) age of the patients was 1.67 (0.92–3.00) years old and the guardians had a median age of 31.0 (28.0–35.0) years. Similar to the bronchiolitis patient group, the majority of pneumonia patients were boys (52.4%) and had no medical insurance (49.3%). Correspondingly, most guardians were female (57.8%), mothers (53.7%), married (98.1%), self-employed or enterprise workers (40.7%), Han nationality (89.2%), urban residents (46.2%) and not religious (96.8%). A total of 40.8% (216/529) had a bachelor or higher, and 63.6% (215/338) earned over 10,000 CNY per month. With the exception of marital status (*p* = 0.373), there were statistically significant differences in other variables between the groups (Table 2).

**Table 2:** Sociodemographic characteristics of the children with pneumonia and their guardians in three regions.

Characteristics	Total	Shanghai	Zhengzhou	Kunming	*p-*value^a^
	*N* = 529	*N* = 173	*N* = 120	*N* = 236	
Sex					0.001
Boys	277 (52.4%)	72 (41.6%)	63 (52.5%)	142 (60.2%)	
Girls	252 (47.6%)	101 (58.4%)	57 (47.5%)	94 (39.8%)	
Age (year)					<0.001
Mean (SD)	2.16 (1.90)	1.67 (1.20)	2.70 (1.19)	2.25 (2.46)	
Median (IQR)	1.67 (0.92–3.00)	1 (0.90–2.00)	2.50 (2.10–3.50)	1.50 (0.40–2.90)	
Medical insurance	*N* = 527	*N* = 171	*N* = 120	*N* = 236	<0.001
Yes	192 (36.4%)	91 (53.2%)	41 (34.2%)	60 (25.4%)	
No	260 (49.3%)	80 (46.8%)	79 (65.8%)	101 (42.8%)	
Unknown/refuse to answer	75 (14.2%)	0 (0.0%)	0 (0.0%)	75 (31.8%)	
Guardian’s age (year)	*N* = 528	*N* = 172	*N* = 120	*N* = 236	0.009
Mean (SD)	33.39 (9.25)	36.28 (12.63)	32.52 (3.63)	31.73 (7.74)	
Median (IQR)	31.0 (28.0–35.0)	31.0 (28.0–37.0)	32.5 (30.0–35.0)	31.0 (28.0–34.0)	
Guardian’s sex					<0.001
Male	223 (42.2%)	57 (32.9%)	42 (35.0%)	124 (52.5%)	
Female	306 (57.8%)	116 (67.1%)	78 (65.0%)	112 (47.5%)	
Relationship					<0.001
Father	202 (38.2%)	35 (20.2%)	42 (35.0%)	125 (53.0%)	
Mother	284 (53.7%)	105 (60.7%)	78 (65.0%)	101 (42.8.0%)	
Others^b^	43 (8.1%)	33 (19.1%)	0 (0.0%)	10 (4.2%)	
Education level					<0.001
Uneducated	5 (0.9%)	3 (1.7%)	0 (0.0%)	2 (0.8%)	
Primary school	28 (5.3%)	8 (4.6%)	0 (0.0%)	20 (8.5%)	
Junior high school	115 (21.7%)	14 (8.1%)	2 (1.7%)	99 (41.9%)	
Senior high school	96 (18.1%)	30 (17.3%)	15 (12.5%)	51 (21.6%)	
Junior college	69 (13.0%)	23 (13.3%)	25 (20.8%)	21 (8.9%)	
University and above	216 (40.8%)	95 (54.9%)	78 (65.0%)	43 (18.2%)	
Marriage status					0.373
Single	8 (1.5%)	2 (1.2%)	0 (0.0%)	6 (2.5%)	
Married	519 (98.1%)	170 (98.3%)	120 (100.0%)	229 (97.0%)	
Separated	2 (0.4%)	1 (0.6%)	0 (0.0%)	1 (0.4%)	
Employment	*N* = 528	*N* = 172	*N* = 120	*N* = 236	<0.001
Enterprise/self-employed	215 (40.7%)	77 (44.8%)	83 (69.2%)	55 (23.3%)	
Full-time student	1 (0.2%)	1 (0.6%)	0 (0.0%)	0 (0.0%)	
Government/institution employee	86 (16.3%)	20 (11.6%)	24 (20.0%)	42 (17.8%)	
Full-time stay at home/housewife	80 (15.1%)	37 (21.5%)	5 (4.2%)	38 (16.1%)	
Retire	21 (4.0%)	18 (10.5%)	0 (0.0%)	3 (1.3%)	
Farming	58 (11.0%)	11 (6.4%)	2 (1.7%)	45 (19.1%)	
Temporary worker	42 (7.9%)	1 (0.6%)	4 (3.3%)	37 (15.7%)	
Unemployed	22 (4.2%)	7 (4.1%)	2 (1.7%)	13 (5.5%)	
Unknown/refuse to answer	3 (0.6%)	0 (0.0%)	0 (0.0%)	3 (1.3%)	
Monthly Income (CNY)	*N* = 528	*N* = 172	*N* = 120	*N* = 236	<0.001
<5,000	93 (17.6%)	0 (0.0%)	3 (2.5%)	90 (38.1%)	
5,000-10,000	103 (19.5%)	0 (0.0%)	20 (16.7%)	83 (35.2%)	
10,000-20,000	81 (15.3%)	9 (5.2%)	47 (39.2%)	25 (10.6%)	
20,000-30,000	76 (14.4%)	37 (21.5%)	34 (28.3%)	5 (2.1%)	
30,000-50,000	70 (13.2%)	54 (31.4%)	13 (10.8%)	3 (1.3%)	
>50,000	48 (9.1%)	40 (23.3%)	3 (2.5%)	5 (2.1%)	
Unknown/refuse to answer	57 (10.8%)	32 (18.6%)	0 (0.0%)	25 (10.6%)	
Guardian’s ethnicity					<0.001
Han	472 (89.2%)	171 (98.8%)	118 (98.3%)	183 (77.5%)	
Others	57 (10.8%)	2 (1.2%)	2 (1.7%)	53 (22.5%)	
Residence type	*N* = 528	*N* = 172	*N* = 120	*N* = 236	<0.001
City	244 (46.2%)	102 (59.3%)	95 (79.2)	47 (19.9%)	
Town	103 (19.5%)	43 (25.0%)	20 (16.7)	40 (16.9%)	
Rural	180 (34.1%)	27 (15.7%)	5 (4.2)	148 (62.7%)	
Unknown/refuse to answer	1 (0.2%)	0 (0.0%)	0 (0.0%)	1 (0.4%)	
Religion status	*N* = 526	*N* = 170	*N* = 120	*N* = 236	0.001
No	509 (96.8%)	170 (100.0%)	118 (98.3%)	221 (93.6%)	
Yes	14 (2.7%)	0 (0.0%)	1 (0.8%)	13 (5.5%)	
Unknown/refuse to answer	3 (0.6%)	0 (0.0%)	1 (0.8%)	2 (0.8%)	

SD, standard deviations; IQR, interquartile range; CNY: Chinese Yuan. ^a^Comparison of population characteristics among the three regions. Categorical variables and continuous variables were analyzed using chi-square test and Kruskal–Wallis *H* test, respectively. ^b^This refers to a guardian such as a non-parental relative of the affected child.

### Economic burden of disease

The cost data for patients with bronchiolitis are presented in Table 3. The mean (SD) total cost per patient was 5,748 (4,165) CNY, with notably higher costs observed in Kunming (9,688 CNY) compared to Shanghai (3,531 CNY) and Zhengzhou (5,880 CNY). The hospitalization cost averaged 4,162 (1,909) CNY, with the patients from Zhengzhou reporting the highest hospitalization cost (4,834 CNY) and those from Shanghai reporting the lowest (3,529 CNY). The mean transportation cost was 63 (186) and the children in Zhengzhou (108 CNY) had the highest cost. All costs for patients in Shanghai are the lowest. The average length of stay was 4.7 (2.7) days, and the patients from Kunming reported the shortest hospitalization stays (4.0 days). Consequently, the guardians in Kunming reported the highest average hours of work lost (92.4 h) and the highest indirect cost, totaling 4,141 CNY.

**Table 3:** Economic burden of disease in children with bronchiolitis per person.

	Total	Shanghai	Zhengzhou	Kunming	*Post hoc* analysis^b^	*p*-value^a^
	*N* = 338	*N* = 128	*N* = 120	*N* = 90		
Traffic cost (CNY)					ZZ > KM > SH	<0.001
Mean (SD)	63 (186)	2 (5)	108 (203)	90 (259)		
Median (IQR)	8 (0–38)	0 (0–0)	38 (21–73)	10.5 (0–51)		
Range	0–2,110	0–21	0–844	0–2,110		
Hospitalization cost (CNY)	*N* = 321	*N* = 128	*N* = 120	*N* = 73	ZZ > SH,KM > SH	<0.001
Mean (SD)	4,162 (1,909)	3,529 (1,327)	4,834 (2,211)	4,638 (1,608)		
Median (IQR)	3,692 (2743–5,570)	3,076 (2,213-4,162)	4,806 (2,954-6,154)	3,639 (3,639-5,728)		
Range	957–13,467	957–6,442	1,055-10,738	1,841-13,467		
Out-of-pocket cost (CNY)	*N* = 322	*N* = 126	*N* = 120	*N* = 76	KM > SH,KM > ZZ	<0.001
Mean (SD)	2,104 (978)	1,920 (776)	2,086 (1,059)	2,438 (1,066)		
Median (IQR)	2,063 (1,414-2,104)	1,898 (1,307-2,366)	2,023 (1,266-2,680)	2,479 (2,426-2,479)		
Range	436–9,494	522–4,862	436–5,275	633–9,494		
Length of stay (day)	*N* = 337	*N* = 128	*N* = 120	*N* = 89	ZZ > SH,ZZ > KM	<0.001
Mean (SD)	4.7 (2.7)	4.4 (1.4)	5.5 (2.0)	4.0 (4.2)		
Median (IQR)	5 (3–6)	4 (3–5)	5 (4–7)	3 (1–6)		
Range	0–26	2–9	3–10	0–26		
Loss of work time (hour)	*N* = 331	*N* = 128	*N* = 120	*N* = 83	ZZ > SH,KM > SH	<0.001
Mean (SD)	33.1 (70.7)	6.0 (4.1)	20.9 (10.7)	92.4 (122.0)		
Median (IQR)	11 (8–24)	8 (0–9)	20 (12–26)	48 (12–144)		
Range	0–720	0–10	6–60	0–720		
Indirect cost (CNY)^c^	*N* = 331	*N* = 128	*N* = 120	*N* = 83	ZZ > SH,KM > SH	<0.001
Mean (SD)	1,483 (3,166)	269 (181)	938 (478)	4,141 (5,484)		
Median (IQR)	493 (359–1,076)	359 (0–403)	896 (538–1,165)	2,151 (538–6,454)		
Range	0–32,268	0–448	269–2,689	0–32,268		
Total cost (CNY)^d^	*N* = 316	*N* = 128	*N* = 120	*N* = 68	KM > ZZ > SH	<0.001
Mean (SD)	5,748 (4,165)	3,531 (1,352)	5,880 (2,476)	9,688 (6,482)		
Median (IQR)	4,723 (3,218-6,904)	3,335 (2,505-4,444)	5,995 (3,863-7,611)	9,017 (6,151-11,198)		
Range	957–38,280	957 (6,867)	1,514 (12,297)	1,847 (38,280)		

SD, Standard deviations; IQR, Interquartile range; SH, Shanghai; ZZ, Zhengzhou; KM, Kunming; CNY, Chinese Yuan. ^a^Comparison using kwAllPairsDunn test by Bonferroni correction. ^b^Calculated using Kruskal–Wallis H test. ^c^Indirect cost: loss of work hour multiples income per hour that converted from annual average income in China 2022 (8 h per work day, and 250 work days per year) and adjusted into 2023 currency. ^d^Total cost includes hospitalization cost, traffic cost and indirect cost. All cost data were adjusted to 2023 Chinese Yuan (CNY) using China’s annual consumer price index.

The costs associated with pneumonia patients are detailed in Table 4. The average total, transportation, hospitalization, indirect costs, length of stay and loss of work time amounted to 7,783 (7,170) CNY, 118 (280) CNY, 6,096 (5,610) CNY, 2,597 (5,042) CNY, 8.1 (6.8) days and 34.9 (79.0) hours, respectively. In terms of total and hospitalization costs, the patients in Zhengzhou suffered the heaviest economic burden. The mean transportation and indirect costs of patients in Kunming were considerably higher than those in other regions. Similarly, the patients in Shanghai incurred the lowest costs across various categories. The patients in Kunming endured the lengthiest hospitalization period, with their guardians experiencing the highest amount of lost work hours and incurring the highest indirect costs.

**Table 4:** Economic burden of disease in children with pneumonia per person.

	Total	Shanghai	Zhengzhou	Kunming	*Post hoc* analysis^b^	*p*-value^a^
	*N* = 529	*N* = 173	*N* = 120	*N* = 236		
Traffic cost (CNY)					KM > ZZ > SH	<0.001
Mean (SD)	118 (280)	5 (10)	78 (148)	221 (379)		
Median (IQR)	21 (0–158)	0 (0–0)	37 (21–67)	158 (15–211)		
Range	0–3,692	0–63	0–835	0–3,692		
Hospitalization cost (CNY)	*N* = 528	*N* = 172	*N* = 120	*N* = 236	ZZ > KM > SH	<0.001
Mean (SD)	6,096 (5,610)	3,267 (1,554)	8,807 (4,029)	6,780 (7,108)		
Median (IQR)	4,958 (3,326-6,968)	3,067 (2,166-3,977)	8,016 (5,622-11,140)	4,958 (4,958-6,857)		
Range	316–84,393	1,040-8,707	3,165-19,305	316–84,393		
Out-of-pocket cost (CNY)	*N* = 521	*N* = 165	*N* = 120	*N* = 236	ZZ > KM > SH	<0.001
Mean (SD)	4,059 (4,153)	2,147 (1,136)	3,875 (2,022)	5,490 (5,527)		
Median (IQR)	3,362 (196–4,220)	1,920 (1,360-2,574)	3,186 (2,584-4,672)	3,903 (3,903-5,802)		
Range	497–63,295	497–8,681	1,371-10,585	527–63,295		
Length of stay (day)					ZZ > SH,KM > SH	<0.001
Mean (SD)	8.1 (6.8)	4.8 (1.9)	8.2 (3.0)	10.5 (9.0)		
Median (IQR)	6 (5–9)	4 (3–6)	7 (6–10)	8 (6–11)		
Range	0–50	1–11	5–15	0–50		
Loss of work time (hour)					ZZ > SH > KM	<0.001
Mean (SD)	34.9 (79.0)	4.8 (4.4)	33.1 (16.1)	57.9 (112.0)		
Median (IQR)	9 (0–40)	8 (0–9)	30 (20–42)	0 (0–100)		
Range	0–1,200	0–10	8–80	0–1,200		
Indirect cost (CNY)^c^					ZZ > SH > KM	<0.001
Mean (SD)	1,565 (3,538)	215 (197)	1,483 (721)	2,597 (5,042)		
Median (IQR)	403 (0–1,793)	359 (0–403)	1,345 (896–1,893)	0 (0–4,482)		
Range	0–53,780	0–448	359–3,585	0–53,780		
Total cost (CNY)^d^	*N* = 528	*N* = 172	*N* = 120	*N* = 236	ZZ > KM > SH	<0.001
Mean (SD)	7,783 (7,170)	3,488 (1,597)	10,367 (4,433)	9,598 (9,125)		
Median (IQR)	5,522 (4,051-9,583)	3,333 (2,271-4,251)	9,606 (6,688-13,723)	6,364 (5,116-10,657)		
Range	1,040–84,393	1,040-9,208	3,955-21,565	2,468-84,393		

SD, Standard deviations; IQR, Interquartile range; SH, Shanghai; ZZ, Zhengzhou; KM, Kunming; CNY, Chinese Yuan. ^a^Comparison using kwAllPairsDunn test by Bonferroni correction. ^b^Calculated using Kruskal–Wallis *H* test. ^c^Indirect cost: loss of work hour multiples income per hour that converted from annual average income in China 2022 (8 h per work day, and 250 work days per year) and adjusted into 2023 currency. ^d^Total cost includes hospitalization cost, traffic cost and indirect cost. All cost data were adjusted to 2023 Chinese Yuan (CNY) using China’s annual consumer price index.

Moreover, we compared the hospitalization costs and total costs of two groups of patients, and the results showed that the hospitalization costs and total costs of pneumonia patients were significantly higher than those of bronchiolitis patients (*p* < 0.001).

### Regression analysis

The variance inflation factor diagnostic shows that the variance inflation factor is significantly less than 10, indicating no collinearity between the independent variables. Therefore, all variables can be included in the model (20).

In terms of bronchiolitis, a total of 315 subjects were included after excluding missing values (Table 5). The outcomes of the multiple linear regression model indicated a noteworthy increase in total cost for patients from Zhengzhou and Kunming compared to those from Shanghai. Furthermore, compared to the children under the guardianship of fathers, those under mothers or other guardians showed a significantly lower total cost. Additionally, longer periods of work time lost, extended hospital stays, and having medical insurance were all significantly associated with higher total costs.

**Table 5:** Multiple regression analysis results of significant factors of the total cost and hospitalization costs in children with bronchiolitis (*N* = 315).

	Coefficient	95% CI		*p*-value^b^	Coefficient	95% CI		*p*-value^b^
		LCI	UCI			LCI	UCI	
		Total cost^a^				Hospitalization cost		
Region (Shanghai^c^)								
Zhengzhou	0.43	0.33	0.54	<0.001	0.35	0.23	0.47	<0.001
Kunming	0.45	0.26	0.64	<0.001	0.15	−0.06	0.37	0.156
Guardian’s age (year)	–	–	–	–	0.01	0	0.02	0.047
Guardian’s sex (male)								
Female	0.32	−0.01	0.64	0.058	0.37	−0.02	0.77	0.065
Relationship (father^c^)								
Mother	−0.43	−0.77	−0.09	0.012	−0.44	−0.84	−0.03	0.035
Others	−0.28	−0.49	−0.07	0.009	−0.45	−0.82	−0.09	0.015
Marriage status (single^c^)	–	–	–	–				
Married	–	–	–	–	−0.34	−0.72	0.04	0.078
Separated	–	–	–	–	−0.02	−0.54	0.50	0.946
Widowhood	–	–	–	–	−0.68	−1.27	−0.09	0.024
Unknown/refuse to answer	–	–	–	–	−0.05	−0.64	0.54	0.858
Guardian’s ethnicity (Han^c^)					–	–	–	–
Others	−0.18	−0.42	0.05	0.129	–	–	–	–
Loss of work time (hour)	0	0	0	<0.001	0	0	0	0.037
Medica insurance (yes^c^)								
No	−0.23	−0.33	−0.13	<0.001	−0.23	−0.33	−0.12	0.001
Unknown/refuse to answer	0.11	−0.07	0.3	0.229	0.23	0.02	0.44	0.033
Length of stay (Day)	0.07	0.05	0.09	<0.001	0.08	0.06	0.1	<0.001
Constant	7.94	7.81	8.06	<0.001	7.79	7.33	8.25	<0.001
*R*-square	0.624				0.319			

CI, confidence interval. ^a^Total cost includes hospitalization costs, traffic costs and indirect costs. ^b^Calculated using multiple linear regression. ^c^Reference category.

Regarding hospitalization costs in bronchiolitis patients, it was observed that older children with medical insurance, residing in Zhengzhou, and experiencing longer hospital stays were more likely to have higher hospitalization costs. Conversely, children under the care of mothers or other guardians, those who lost less work time and were widowhood were associated with lower hospitalization costs (Table 5).

Regression models were also conducted to analyze the correlation between sociodemographic characteristics and both total costs and hospitalization costs in pneumonia patients (Table 6). The results indicated a significant association between total cost and factors such as region, medical insurance, loss of work time, and length of stay. Similarly, hospitalization costs showed a significant correlation with region, medical insurance, guardian’s age, employment, loss of work time, and length of stay.

**Table 6:** Multiple regression analysis results of significant factors of the total cost and hospitalization costs in children with pneumonia (*N* = 522).

	Coefficient	95% CI		*p-*value^b^	Coefficient	95% CI		*p-*value^b^
		LCI	UCI			LCI	UCI	
	Total cost^a^				Hospitalization cost			
Region (Shanghai^c^)								
Zhengzhou	0.99	0.89	1.09	<0.001	0.96	0.82	1.1	<0.001
Kunming	0.9	0.8	1	<0.001	0.58	0.44	0.72	<0.001
Sex (Boys^c^)								
Girls	–	–	–	–	−0.09	−0.19	0.01	0.068
Guardian’s age (year)	–	–	–	–	−0.01	−0.01	0	0.046
Education level (uneducated^c^)								
Primary school	0.14	−0.25	0.53	0.479	−0.05	−0.58	0.48	0.860
Junior high school	−0.08	−0.45	0.29	0.682	−0.35	−0.87	0.18	0.192
Senior high school	0.02	−0.34	0.39	0.904	−0.37	−0.9	0.16	0.166
Junior college	0	−0.37	0.37	0.997	−0.4	−0.93	0.14	0.146
University and above	0.1	−0.26	0.46	0.59	−0.26	−0.79	0.27	0.336
Employment (Enterprise/self-employed^c^)								
Full-time student	–	–	–	–	−0.22	−1.3	0.86	0.692
Government/institution employee	–	–	–	–	−0.2	−0.34	−0.05	0.008
Full-time stay at home/housewife	–	–	–	–	0.09	−0.06	0.25	0.241
Retire	–	–	–	–	0.21	−0.12	0.54	0.215
Farming	–	–	–	–	−0.15	−0.35	0.04	0.128
Temporary worker	–	–	–	–	−0.38	−0.58	−0.18	<0.001
Unemployed	–	–	–	–	0.12	−0.12	0.37	0.326
Unknown/refuse to answer	–	–	–	–	0.12	−0.51	0.75	0.712
Loss of work time (hour)	0	0	<0.001	<0.001	–	–	–	–
Medical insurance (yes^c^)								
No	−0.19	−0.27	−0.11	<0.001	−0.25	−0.36	−0.14	<0.001
Unknown/refuse to answer	−0.63	−0.77	−0.49	<0.001	−0.46	−0.65	−0.27	<0.001
Length of stay (day)	0.02	0.02	0.03	<0.001	0.04	0.03	0.04	<0.001
Constant	7.96	7.60	8.32	<0.001	8.52	7.88	9.16	<0.001
*R*-square	0.654				0.424			

CI, confidence interval. ^a^Total cost includes hospitalization costs, traffic costs and indirect costs. ^b^Calculated using multiple linear regression. ^c^Reference category.

## Discussion

This study conducted face-to-face interviews with caregivers of bronchiolitis and pneumonia pediatric patients from three distinct socioeconomic regions in China. The analysis provided a comprehensive exploration of costs and identified significant factors contributing to variations in these costs. The results revealed that the total cost per bronchiolitis patient across different regions ranged from 957 to 38,280 CNY. For pneumonia patients, the range was broader, from 1,040 to 84,393 CNY, with hospitalization expenses being the primary contributor. Key factors significantly influencing the economic burden were also identified through the study.

In this study, we examined the comprehensive economic burden of diseases, encompassing transportation costs, hospitalization expenses, and the indirect costs associated with lost working time. Our findings revealed that the average hospitalization cost and duration for children under the age of 5 with bronchiolitis in China were slightly lower than previously reported figures. Notably, in Wang et al.’s (11) study, the costs were adjusted to 2023 using the CPI, indicating an average hospitalization cost of 7,470 CNY and a length of stay of 8.5 days in Gansu Province. This might be attributed to the lower economic level of Gansu Province in comparison to the three regions examined in this study, resulting in a relatively lower standard of healthcare and, consequently, a higher economic burden. This finding aligned with the results in this study, which suggested that individuals residing in regions with lower economic levels experienced a greater economic burden. When comparing our study with previous investigations exploring the economic burden of pneumonia patients in China, the total hospitalization costs were marginally lower than those reported by Li et al. (8) and slightly higher than those presented by Wang et al. (14).

The average total cost associated with bronchiolitis and pneumonia represented approximately 6.71 and 9.08% of China’s *per capita* GDP (85,686 CNY), respectively, imposing a substantial financial strain on households (21). Notably, the economic burden of these diseases exhibited significant regional variations. The latest *per capita* GDP in Shanghai, Zhengzhou, and Kunming stood at 180,400 CNY, 101,528 CNY, and 88,701 CNY, respectively (21). In alignment, the total cost of bronchiolitis accounted for 1.96, 5.80, and 10.92% of the local *per capita* GDP, while the total cost of pneumonia constituted 1.93, 10.21, and 10.81%, respectively. These findings underscore that both bronchiolitis and pneumonia place a more formidable economic burden on families in economically underdeveloped areas. As a response, the government could implement necessary policies, such as improving the implementation of medical insurance and assistance systems, to alleviate the financial strain on families.

Whether it was bronchiolitis or pneumonia, the percentage of children without medical insurance exceeded the average coverage level of basic medical insurance in Chinese society. This may be attributed to some families in China do not purchase medical insurance specifically for their children. Additionally, the basic medical insurance system is significantly constrained by the local economic development level, leading to variations in insurance coverage across different levels of economic development in this study (14). Whether it is bronchitis or pneumonia, Kunming exhibits the lowest rate of patients with evident medical insurance coverage. Approximately 68.9% of bronchitis patients in the area either remain uncertain or decline to disclose their medical insurance status. This phenomenon could be attributed to the comparatively low economic income level in the region, thereby limiting patients’ capacity or awareness to acquire medical insurance. Moreover, children without medical insurance reported lower hospitalization and overall costs. A previous study indicated that relying solely on out-of-pocket expenses as a means of accessing treatment poses barriers, making individuals seeking treatment more inclined toward those who can afford medical expenses (22).

The regression results consistently indicated the total and hospitalization costs exhibited a positive correlation with the duration of hospitalization, which was consistent with previous studies showing an increase trend in the economic burden of hospitalization days (23). Patients in Shanghai incurred the lowest hospitalization costs and total expenses, potentially owing to the superior medical technology and better baseline health status of individuals in Shanghai. Notably, patients in Kunming records the highest transportation and indirect costs. This disparity could be attributed to the comparatively lower socio-economic development level in Kunming. Despite this, medical resources in other cities within Yunnan Province were even scarcer. Consequently, patients living outside Kunming in Yunnan were inclined to seek better treatment by traveling to Kunming, resulting in an increasing loss of work time (24).

This study has several limitations. First, the inclusion of hospitalized patients only restricts the ability to effectively reflect the economic burden associated with alternative treatment methods, such as outpatient care. This limitation might impact the representativeness of the study population and the generalizability of the research results. Second, some expenses were predominantly reported through guardians recalling treatment-related costs, introducing the possibility of recall bias. Third, the absence of data on specific clinical characteristics, such as disease severity and treatment methods, prevents the assessment of their impact on the economic burden. What’s more, some variable results in this study have missing values, which could influence the outcomes by excluding this data in regression analysis. Fifth, due to constraints in research design and resources, we did not include healthy children as a control group for comparison and did not conduct long-term follow-up. Sixth, regression model validation was not conducted due to a lack of data. Last, due to the reliance on patient reports for cost collection in this study, and the lack of a detailed collection of each expenditure item, a thorough analysis of the specific factors contributing to cost variations in each region may not be feasible.

## Conclusion

Despite limitations such as recall bias and missing data, this study still found that the economic burden of both bronchiolitis and pneumonia is significant in China. And the economic burden varies across regions, imposing a particularly heavy toll on families in underdeveloped areas. Notably, the economic burden on pneumonia patients surpasses that of bronchiolitis. Therefore, it is necessary to develop targeted measures to reduce medical costs and increase insurance coverage, especially in economically disadvantaged areas.

## Data Availability

The raw data supporting the conclusions of this article will be made available by the authors, without undue reservation.
